# Dynapenia could predict chemotherapy-induced dose-limiting neurotoxicity in digestive cancer patients

**DOI:** 10.1186/s12885-018-4860-1

**Published:** 2018-10-04

**Authors:** Damien Botsen, Marie-Amélie Ordan, Coralie Barbe, Camille Mazza, Marine Perrier, Johanna Moreau, Mathilde Brasseur, Yohann Renard, Barbara Taillière, Florian Slimano, Eric Bertin, Olivier Bouché

**Affiliations:** 1 Ambulatory Cancer Unit, Reims University Hospital, Robert Debré Hospital, rue du Général Koenig, 51100 Reims, France; 2 Clinical Research Unit, Reims University Hospital, Robert Debré Hospital, rue du Général Koenig, 51100 Reims, France; 3 Department of Gastroenterology and Digestive Oncology, Reims University Hospital, Robert Debré Hospital, rue du Général Koenig, 51100 Reims, France; 4 Department of General and Digestive Surgery, Reims University Hospital, Reims, France; 5 Artificial Nutrition Unit, Reims University Hospital, Robert Debré Hospital, rue du Général Koenig, 51100 Reims, France; 6 Department of Nutrition, Endocrinology, and Diabetology, Reims University Hospital, Robert Debré Hospital, rue du Général Koenig, 51100 Reims, France

**Keywords:** Dynapenia, Antineoplastic agents, Digestive system neoplasms, Sarcopenia, Dose-limiting toxicity, Muscle strength

## Abstract

**Background:**

FIGHTDIGO study showed the feasibility and acceptability of handgrip strength (HGS) measure in routine in 201 consecutive patients with digestive cancer treated with ambulatory chemotherapy. The present study focuses on the second aim of FIGHTDIGO study: the relationships between pre-therapeutic dynapenia and chemotherapy-induced Dose-Limiting Toxicities (DLT).

**Methods:**

In this ancillary prospective study, DLT were analyzed in a sub-group of 45 chemotherapy-naive patients. Two bilateral consecutive measures of HGS were performed with a Jamar dynamometer before the first cycle of chemotherapy. Dynapenia was defined as HGS < 30 kg (men) and < 20 kg (women). DLT and/or Dose-Limiting Neurotoxicity (DLN) were defined as any toxicity leading to dose reduction, treatment delays or permanent treatment discontinuation.

**Results:**

Two-thirds of chemotherapies were potentially neurotoxic (*n* = 31 [68.7%]) and 22 patients (48.9%) received FOLFOX (5FU, leucovorin plus oxaliplatin) regimen chemotherapy. Eleven patients (24.4%) had pre-therapeutic dynapenia. The median number of chemotherapy cycles was 10 with a median follow-up of 167 days. Twenty-two patients experienced DLT (48.9%). There was no significant association between pre-therapeutic dynapenia and DLT (*p* = 0.62). Nineteen patients (42.2%) experienced DLN. In multivariate analysis, dynapenia and tumoral location (stomach, biliary tract or small intestine) were independent risk factors for DLN (HR = 3.5 [1.3; 9.8]; *p = 0.*02 and HR = 3.6 [1.3; 10.0]; *p* = 0.01, respectively).

**Conclusions:**

Digestive cancer patients with pre-therapeutic dynapenia seemed to experience more DLN. HGS routine measurement may be a way to screen patients with frailty marker (dynapenia) who would require chemotherapy dose adjustment and adapted physical activity programs.

**Trial registration:**

NCT02797197 June 13, 2016 retrospectively registered.

## Background

Digestive cancers represent most of cancer spectrum in the world [1] with cytotoxic drugs and targeted biotherapy mainly used. Cancer cachexia is a multifactorial syndrome defined by an ongoing loss of skeletal muscle (with or without loss of fat mass) that cannot be fully reversed by conventional nutritional support and leads to progressive functional impairment (loss of muscle strength) [2]. Sarcopenia is defined as the age-related loss in skeletal muscle mass, and function (dynapenia [3] or performance) [4, 5]. Historically, calculation of anti-cancer treatment dose used body surface area (BSA) formula [6] depending on patient’s weight and height. However, the objective at this time was not to develop a formula to dose anti-cancer drugs. Furthermore, body composition has emerged as an important predictor of anti-cancer drug efficacy and toxicity [7–13]. In 2013, a systematic review led by Prado et al. has described a correlation between low lean tissue and severe toxicities of antineoplastic agents due to plasmatic surexposure [14]. A growing literature suggests that the lean tissue compartment may be used to adjust dosage of drugs that are distributed in and metabolized by lean tissues [14–17]. Loss of muscle mass occurs in 80% of patients with cancer [18] but cannot be interpreted without its function, that is muscle strength.

Handgrip Strength (HGS) has been validated in geriatric and surgical studies. In oncology, HGS is associated with cancer-related fatigue, poor quality of life, loss of functional status in hospitalized patients, postoperative complications, length of hospital stay and short term survival [19–23]. It has also been included in the consensual definition of sarcopenia, which associates muscle mass loss and weak strength [5]. Muscle mass is one of the determinants of muscle strength [24], but needs invasive and costly exams to be estimated (X-ray absorptiometry(DXA), anthropometry and bioelectrical impedance analysis (BIA), magnetic resonance imaging (MRI) or computerized tomography (CT)). HGS may be a low cost method to detect sarcopenia in clinical settings and by extrapolation to predict anticancer drug toxicity in patients with advanced cancers [25]. Recently, Ordan et al. [23] showed in FIGHTDIGO study the feasibility and acceptability of HGS measure with a Jamar dynamometer in routine. The present study focuses on the second aim of FIGHTDIGO study: the association between pre-therapeutic dynapenia and chemotherapy-induced Dose-Limiting Toxicities (DLT) in digestive cancer patients treated with ambulatory chemotherapy.

## Methods

### Study design and participants

This ancillary prospective monocentric study was conducted in the Ambulatory Cancer Unit (UMA-CH) of the Reims teaching hospital in France. The study population of FIGHTDIGO included patients older than 18 years-old, having a primary digestive cancer regardless of stage, and undergoing cytotoxic chemotherapy and/or biotherapy for digestive system cancers. All cytotoxic chemotherapy and/or biotherapy regimens according to guidelines were allowed. Patients who could not give their consent, did not understand the handgrip test, had history of neuro-muscular disorder and/or had appointed a health care proxy were excluded. The patients were recruited from May 18, 2016, to November 18, 2016, and were followed for 6 months. For this study on chemotherapy-induced DLT, in order to avoid an overestimation of toxicities in pre-treated patients, only the sub-group of chemotherapy-naïve patients were included.

### Ethical approval

Informed written consent was obtained for each enrolled patient in the trial. The FIGHTDIGO study was approved by the ethics committee (Committee for the Protection of Person EST I DIJON, 25 March 2016) and was registered in Clinicaltrials.gov (NCT02797197).

### Outcome

The aim was to evaluate pre-therapeutic dynapenia as a predictive factor of chemotherapy-induced DLT and/or Dose-Limiting Neurotoxicity (DLN). Other potential predictive factors were analyzed (patient, tumoral, nutritional (as modified Glasgow Prognostic Score (mGPS)) and therapeutic characteristics).

### Handgrip strength (HGS) measurement

HGS was measured, at first hospital stay, before chemotherapy administration, in non-dominant and dominant hand using a hydraulic Jamar Dynamometer. Position 2 was used among the 5 possible handle-positions. Patients performed the test while sitting comfortably (feet touching ground) with shoulder adducted, forearm neutrally rotated and elbow flexed to 90°. The other upper limb was placed alongside the body and relaxed. Patients were instructed (by physicians) to perform maximal isometric contraction within 3 s in both hand. Four measurements were taken. Two measurements were determined for each hand alternatively. A one-minute break was respected between two measurements with the same hand. The highest value (from the four measurements obtained) was chosen for final evaluation. According to the European Working Group on Sarcopenia, dynapenia was defined as HGS < 30 kg (men) and < 20 kg (women) [5].

### Chemotherapy-induced dose-limiting toxicities (DLT) and dose-limiting neurotoxicity (DLN)

Data were prospectively recorded in medical file at each hospital stay. Chemotherapy-induced DLT (all non-neurological toxicities) were defined as any toxicity leading to dose reduction (temporary or permanent), treatment delays or permanent treatment discontinuation. Chemotherapy-induced DLN was defined as permanent peripheral neuropathy (Levi scale grade 2 or 3) leading to dose reduction or permanent treatment discontinuation. DLN and progressive disease as the cause of treatment termination were not considered as DLT. Pre-therapeutic dose adaptation was defined as an initial dose reduction by individual clinical appreciation taking into account patient profile (age, ECOG Performance Status (PS), organ failure, malnutrition or mGPS). Malnutrition was defined as BMI < 21 kg/m^2^ in patients aged more than 70 years old and BMI < 18.5 kg/m^2^ in patients aged less than 70 years old [26].

### Statistical analysis

Data were described using mean ± standard deviation for quantitative variables and numbers (percentage) for qualitative variables.

The survival curves were established by the Kaplan-Meier method. Prognostic factors were identified by univariate analysis using the log rank tests and by multivariate analysis using a Cox proportional hazard model. Factors significant at the 0.20 level in univariate analysis were included in a stepwise regression multivariate analysis with entry and removal limits set at 0.20. Concerning analysis of factors associated with chemotherapy-induced DLN, neurotoxic chemotherapy was not included in multivariate analysis because of one strata has no event (convergence wasn’t satisfied) and BMI was not included in multivariate analysis because of multicollinearity. Comparisons between subgroups of patients were performed using Chi 2 test, Fisher’s exact test, Student’s t test or Wilcoxon test, as appropriate.

All analyses were performed using SAS version 9.4 (SAS Institute Inc., Cary, NC, USA).

## Results

### Description of the population

Among the 201 consecutive patients of the FIGHTDIGO study, 45 were chemotherapy-naïve. Characteristics of those 45 patients are presented in Table 1. Mean age was 66.2 ± 12.3 years. Colorectal cancer was the most frequent digestive location (*n* = 22 [51.2%]). The majority of participants were treated for localized tumor (*n* = 20 [44.4%]). Median follow up was 167 days [0–189], corresponding to a median number of 10 [0–18] ambulatory hospitalizations for chemotherapy. Eighty-four percent (*n* = 38) of the patients underwent chemotherapy alone and 16% (*n* = 7) underwent combination of chemotherapy and biotherapy. The majority of anticancer drugs were potentially neurotoxic (*n* = 31 [68.7%]) and the most frequently received chemotherapy regimen was FOLFOX (5FU, leucovorin plus oxaliplatin). Fourteen patients (31.1%) were overweight (BMI > 25 kg/m^2^). Eleven patients (24.4%) had dynapenia.

**Table 1:** Baseline characteristics (*n* = 45)

Characteristics^a^	Value
Age, mean ± SD	66.2 ± 12.3
Sex	
Male	21 (46.7)
Female	24 (53.3)
Body Mass Index, mean ± SD, kg/m^2^	23.2 ± 4.1
BMI categories, No. (%)	
Malnutrition ^b^	7 (15.6)
Normal	24 (53.3)
Overweight ^c^	14 (31.1)
ECOG PS, No. (%)	
0	12 (26.7)
1	31 (68.9)
2	2 (4.4)
mGPS^d^, No. (%)	
0	17 (43.6)
1	17(43.6)
2	5 (12.8)
Hospitalizations number, median [range]	10 [0–18]
Follow-up, median [range], days	167 [0–189]
Primary tumor location, No. (%)	
Colon and rectum	22 (48.9)
Esophagus	3 (6.7)
Stomach	5 (11.1)
Biliary tract	1 (2.2)
Pancreas	9 (20.0)
Small intestine	1 (2.2)
Neuroendocrine tumor	2 (4.4)
Unknown	2 (4.4)
Stage, No. (%)	
Local	20 (44.4)
Locally advanced	6 (13.3)
Metastatic	19 (42.2)
Type of treatment, No. (%)	
Chemotherapy	38 (84.4)
Chemotherapy and biotherapy	7 (15.6)
Chemotherapy protocol, No. (%)	
5FU + OXALIPLATIN	22 (48.9)
5FU + IRINOTECAN + OXALIPLATIN	7 (15.6)
5FU alone	6 (13.3)
GEMCITABINE	5 (11.1)
5FU-DACARBAZINE	2 (4.4)
5FU + IRINOTECAN	1 (2.2)
GEMCITABINE + OXALIPLATIN	1 (2.2)
VP16 + CISPLATINE	1 (2.2)
Neurotoxic chemotherapy ^e^, No. (%)	31 (68.9)
Biotherapy protocol, No. (%)	
BEVACIZUMAB	6/7 (85.7)
CETUXIMAB	1/7 (14.3)
Dynapenia, No. (%)	11 (24.4)

*SD* standard deviation, *BMI* body mass index, *mGPS* modified Glasgow Prognostic Score, *ECOG PS* Eastern Cooperative Oncology Group Criteria Performance Status

^a^Data are expressed as No (%) unless otherwise indicated

^b^malnutrition was defined as BMI < 21 kg/m^2^ in patients aged more than 70 years old and BMI < 18.5 kg/m^2^ in patients aged less than 70 years old

^c^overweight was defined as BMI > 25 kg/m^2^

^d^6 missing data

^e^neurotoxic regimens: 5FU + IRINOTECAN+OXALIPLATIN, 5FU + OXALIPLATIN,GEMCITABINE + OXALIPLATIN; VP16-CISPLATINE

### Chemotherapy-induced DLT

Results are shown in Table 2. DLT occurred in 22 patients (48.9%). Most of DLT was digestive (63.6%) (diarrhea (*n* = 8), mucositis (*n* = 4), vomiting (*n* = 2)) and hematological (36.4%) (thrombopenia (*n* = 4), neutropenia (*n* = 3) and febrile neutropenia (*n* = 1)). None characteristic was significantly associated with DLT in univariate analysis. There was no significant association between dynapenia and DLT (*n* = 7 (31.8%) in DLT group versus *n* = 4 (17.4%); *p* = 0.62).

**Table 2:** Factors associated with chemotherapy-induced Dose-Limiting Toxicities (DLT)

Characteristics^a^	DLTs	No DLT	Univariate analysis
	(*n* = 22)	(*n* = 23)	*p* value
Maximum handgrip strength, mean ± SD	26.1 ± 7.2	31.2 ± 10.8	0.21
Dynapenia	7 (31.8)	4 (17.4)	0.62
Age, mean ±	66.2 ± 13.5	66.3 ± 11.4	0.53
ECOG PS			0.90
0	6 (27.3)	6 (26.1)	
1	16 (72.7)	15 (65.2)	
2	0 (0)	2 (8.7)	
mGPS^b^			0.12
0, N (%)	11 (64.7)	6 (35.3)	
1, N (%)	7 (41.2)	10 (58.8)	
2, N (%)	2 (40.0)	3 (60.0)	
0	11 (55.0)	6 (31.6)	
1	7 (35.0)	10 (52.6)	
2	2 (10.0)	3 (15.8)	
Primary tumor location			0.21
Colon and rectum	12 (57.1)	10 (45.4)	
Esophagus	1 (4.8)	2 (9.1)	
Stomach	1 (4.8)	4 (18.2)	
Biliary tract	1 (4.8)	0 (0)	
Pancreas	6 (28.6)	3 (13.6)	
Small intestine	0 (0)	1 (4.5)	
Neuroendocrine tumor	0 (0)	2 (9.1)	
Stage	22 (48.8)	23 (51.2)	0.77
Local, N (%)	11 (55.0)	9 (45.0)	
Locally advanced, N (%)	3 (50.0)	3 (50.0)	
Metastatic, N (%)	8 (42.1)	11 (57.9)	
Local	11 (50)	9 (39.1)	
Locally advanced	3 (13.6)	3 (13.0)	
Metastatic	8 (36.4)	11 (47.8)	
BMI, mean ± SD, kg/m2	22.7 ± 3.9	23.8 ± 4.3	0.64
BMI categories			0.36
Malnutrition ^c^	5 (22.7)	2 (8.7)	
Normal	11 (50.0)	13 (56.5)	
Overweight^d^	6 (27.3)	8 (34.8)	

*SD* standard deviation, *BMI* body mass index, *mGPS* modified Glasgow Prognostic Score, *ECOG PS* Eastern Cooperative Oncology Group Criteria Performance Status

^a^Data are expressed as No (%) unless otherwise indicated

^b^6 missing data

^c^malnutrition was defined as BMI < 21 kg/m^2^ in patients aged more than 70 years old and BMI < 18.5 kg/m^2^ in patients aged less than 70 years old

^d^overweight was defined as BMI > 25 kg/m^2^

### Chemotherapy-induced DLN

**Fig. 1:**
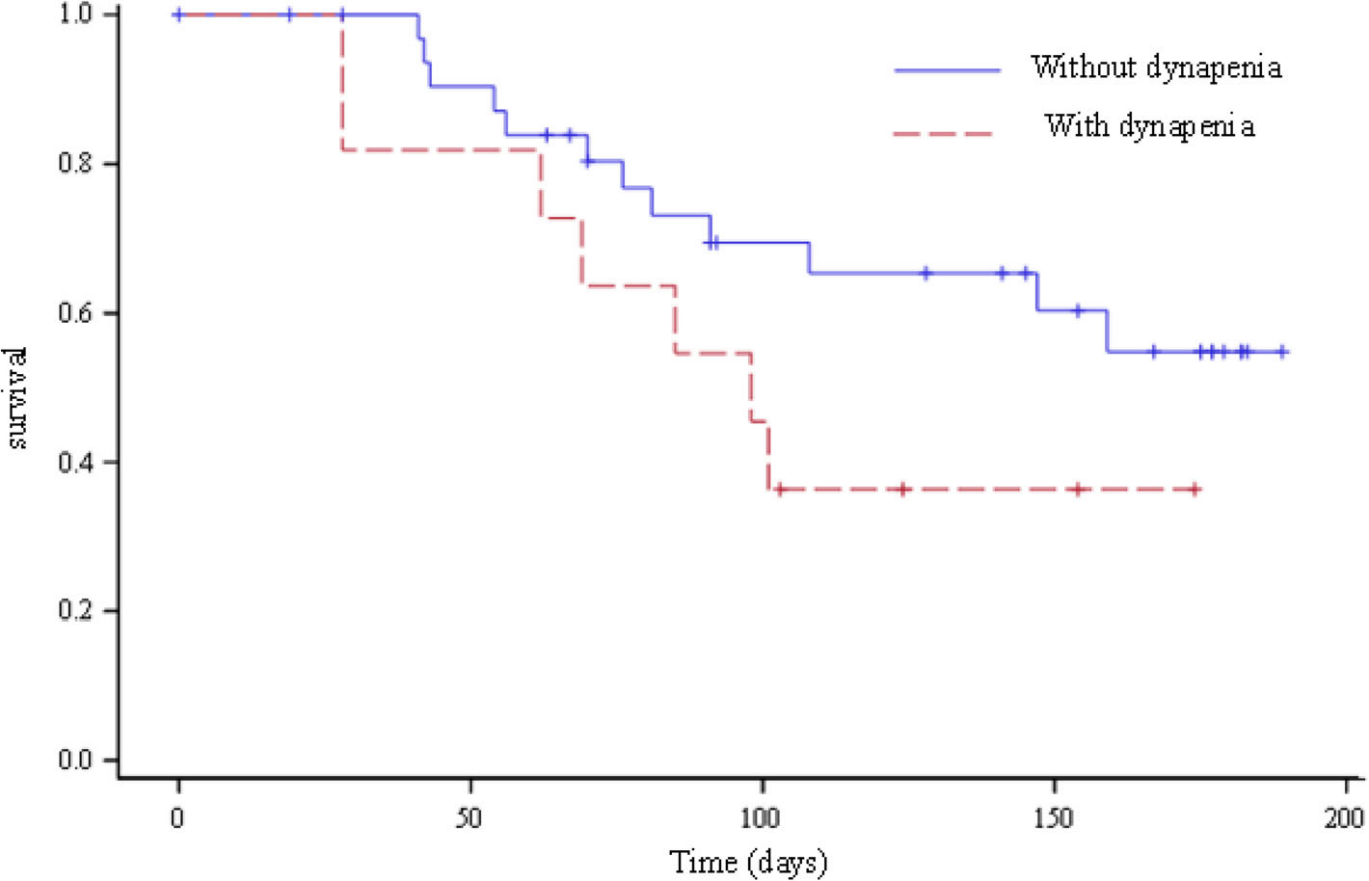
Association between dynapenia and Dose-Limiting Neurotoxicity (DLN). *p* = 0.002. Hazard Ratio = 3.5 [1.3; 9.8]

Results are shown in Table 3. A total of 19 patients experienced DLN (42.2%). Sensitive neuropathy occurred exclusively in patients receiving neurotoxic chemotherapy (*n* = 19 (63.3%) in DLN group versus *n* = 0 (0%) in other patients; *p* < 0.001). Stomach, biliary tract and small intestine cancers received exclusively neurotoxic chemotherapy. In univariate analysis, tumoral location in stomach, biliary tract or small intestine (*p* = 0.02), ECOG PS 1 or 2 (*p* = 0.04), BMI (*p* = 0.048) and overweight (*p* = 0.02) were associated with DLN. No *s*ignificant association with age, mGPS, malnutrition or stage were observed. Four variables were included in multivariate analysis: dynapenia, ECOG PS, overweight and tumoral location (stomach, biliary tract or small intestine). In multivariate analysis, dynapenia and tumoral location were identified as risk factors for DLN (HR = 3.5 [1.3; 9.8]; *p = 0.*02 and HR = 3.6 [1.3; 10.0]; *p* = 0.01, respectively). Association between dynapenia and DLN is shown in Fig. 1. ECOG PS 1 or 2 appeared as a protective factor of DLN (HR = 0.3 [0.1; 0.9]; *p* = 0.03).

**Table 3:** Factors associated with chemotherapy-induced Dose-Limiting Neurotoxicity (DLN)

Characteristics^a^	DLNs	No DLN	Univariate analysis	Multivariate analysis^d^	
	(*n* = 19)	(*n* = 26)	*p* value	HR [95% CI]	*p* value
Maximum handgrip strength, mean ± SD	29.1 ± 10.3	28.5 ± 9.1	0.64		
Dynapenia			0.13		0.02
No	12 (63.2)	22 (84.6)		1	
Yes	7 (36.8)	4 (15.4)		3.5 [1.3; 9.8]	
Age, mean ± SD	65.9 ± 11.5	66.5 ± 13.1	0.89		
ECOG PS			0.04		0.03
0	8 (42.1)	4 (15.4)		1	
1 or 2	11 (57.9)	22 (84.6)		0.4 [0.2; 0.9]	
mGPS ^b^			0.39		
0	8 (50.0)	9 (39.1)			
1	8 (50.0)	9 (39.1)			
2	0 (0.0)	2 (21.7)			
Primary Tumor location			0.02		0.01
Stomach – Biliary tract – Small Intestine	6 (31.6)	1 (4.2)		3.6 [1.3; 10.0]	
Other location	13 (68.4)	23 (95.8)		1	
Stage			0.49		
Local	10 (52.6)	10 (38.5)			
Locally advanced	1 (5.3)	5 (19.2)			
Metastatic	8 (42.1)	11 (42.3)			
BMI, mean ± SD, kg/m2	23.9 ± 3.4	22.8 ± 4.5	0.048		
Overweight, No. (%)^c^			0.02		
Yes	9 (47.4)	5 (19.2)			
No	10 (52.6)	21 (80.8)			

*SD* standard deviation, *HR* Hazard Ratio, *BMI* body mass index, *mGPS* modified Glasgow Prognostic Score, *ECOG PS* Eastern Cooperative Oncology Group Criteria Performance Status

^a^Data are expressed as No (%) unless otherwise indicated

^b^6 missing data

^c^overweight was defined as BMI > 25 kg/m^2^

^d^four variables were included in multivariate analysis: dynapenia, ECOG PS, overweight and tumoral location, neurotoxic chemotherapy was not included in multivariate analysis because of one strata has no event (convergence wasn’t satisfied)

### Pre-therapeutic chemotherapy dose adaptation

Table 4 showed results. In univariate analysis, no significant association between pre-therapeutic dose adaptation and ECOG PS 1 or 2 (*p* = 0.70) or malnutrition (*p* = 0.64) were found. The 2 patients with ECOG PS 2 had a pre-therapeutic adaptation.

**Table 4:** Factors associated with dose adaptation

Characteristics ^a^	Dose adaptation	Dose complete	Univariate analysis
			*p* value
ECOG PS			0.70
0	2 (16.7)	10 (83.3)	
1 or 2	8 (24.2)	25 (75.8)	
Malnutrition	2 (28.6)	5 (71.4)	0.64

^a^Data are expressed as No (%) unless otherwise indicated

*ECOG PS* Eastern Cooperative Oncology Group Criteria Performance Status, *SD* standard deviation; malnutrition was defined as BMI < 21 kg/m^2^ in patients aged more than 70 years old and BMI < 18.5 kg/m^2^ in patients aged less than 70 years old

## Discussion

To our knowledge, this prospective study is the first to show that pre-therapeutic dynapenia (low HGS testing with a Jamar dynamometer) could be predictive of chemotherapy-induced DLN in digestive cancer patients. Majority of participants received a neurotoxic regimen which enhanced these findings. Stomach, biliary tract and small intestine cancers were significantly associated with DLN in unadjusted and adjusted analysis because of receiving exclusively neurotoxic chemotherapy. There was no association of dynapenia with chemotherapy-induced DLT. This might be explained by several elements: efficacy of supportive cares, good safety profile of chemotherapy regimen in digestive cancers (as FOLFOX regimen [27]), favorable prognostic profile of our population [1] and a small sample size. Conversely, in a pilot study in 112 older newly diagnosed cancer patients with a minority of digestive cancer (*n* = 20), low grip strength predicted toxicity at 3 months [28]. In our study, as none significant association of chemotherapy-induced DLT could be detected with age, malnutrition, BMI, mGPS and stage, and ECOG PS 1 or 2 was found to be a protective factor of DLN, heterogeneity of population and pre-therapeutic dose adaptation of chemotherapy might be an explanation of discrepancies. Most of oncology trials excluded patients with ECOG PS > 1 [29–31]. A bad condition at inclusion could be a bias for evaluation and limit the scope of potentially tested treatments. Modified GPS is an inflammation-based prognostic score. It has been shown to be correlated with mortality in colon cancer [32] influenced by malnutrition and predicting toxicity [33]. In present study, a small sample size with mGPS = 2 and/or ECOG PS 2 might be responsible of non-significant results.

Cancer cachexia is a recent concept that has been defined with the emergence of the body composition consideration in oncology [18] [34]. Prado et al. has reported the association of body composition with chemotherapy-induced DLT in stage II-III colon cancer patients treated with 5FU and leucovorin [35]. These results had consequences on phase I studies with many works on relationship between sarcopenia/body composition and DLT [9, 11]. Body composition and sarcopenia seem to be prognostic of enhanced chemotherapy and targeted therapy toxicity [7, 8, 10, 11, 13], and more recently with immune checkpoint inhibitors such as anti-CTLA-4 (ipilimumab) [12].

Lean Body Mass (LBM) was associated with chemotherapy-induced DLT and DLN in patients with colon cancers treated with FOLFOX regimen [36]. Neurotoxicity affects 80% of patients and becomes chronic in 15–20% of cases, sometimes irreversibly [27]. A recent systematic review [37] has reported the impact of acute oxaliplatin-induced neuropathy and necessity of a large prospective study to established better preventive guidelines. In 2 cohorts of colorectal cancer patients treated with oxaliplatin, Ali et al. [36] reported that overall DLT, and specifically oxaliplatin-induced neuropathy, occurred mostly in patients who receive > 3.09 mg/oxaliplatin/kg LBM. One study is ongoing to determine whether the oxaliplatin dose normalization based on the LBM index can prevent or reduce neurotoxicity associated with oxaliplatin, for colorectal cancer patients treated in adjuvant with FOLFOX regimen (LEANOX trial NCT03255434). Our findings might be an additional argument for this hypothesis. Dynapenia, which is the first step toward sarcopenia, may represent an interesting tool to estimate sarcopenia / LBM [38]. It might be a more easily accessible predictive marker for oxaliplatin dose adaptation because patients with low muscle mass behave like patients “overdosed” with chemotherapy resulted in dose-limiting toxicities, independently of the patient’s weight.

In present study, there were 31% of overweight patients. Recently, it has been reported that overweight sarcopenic patients experienced more DLT [12, 39, 40] than overweight patients with normal LBM. A potential explanation could be an altered volume distribution, metabolism or clearance in lean tissue of anticancer drugs in these patients [41]. Dose of cytotoxic drugs has been shown to be correlated with LBM [35]. These findings suggest a new paradigm in the future: body composition measurement leading to dose adaptation of antineoplastic agents.

Gray et al. have reported a prediction model used to identify sarcopenia based on parameters of functional fitness [42]. Correlation between measure of dynapenia and skeletal muscle index by computed tomography in a cancer population are being evaluated in a further ancillary analysis of FIGHTDIGO study.

HGS measure is a useful low-cost method [23] which could improve clinical practice by stratifying frailty patients who experienced more DLN. More relevant nutritional and adapted physical activity programs might be needed.

A major limitation of this ancillary study is the size of cohort with only 45 patients with various type of digestive cancers and heterogeneous chemotherapy regimens. Several aspects of using the Jamar dynamometer may require further investigation. Many studies are attempting to determine cut points for dynapenia with respect to age, sex, and ethnic group [5, 43, 44]. The HGS dynamometer thresholds remain undetermined for cancer patients.

## Conclusions

In conclusion, digestive cancer patients with pre-therapeutic dynapenia seemed to experience more chemotherapy-induced DLN. This small cohort study suggests that dynapenia could be a predictive marker of chemotherapy-induced DLN. Further studies need to be performed to obtain more definitive data. Since dynapenia is the first step toward sarcopenia, HGS routine measurement may be a way to screen patients with frailty marker who require dose adaptation of antineoplastic agents and adapted physical activity programs to prevent chemotherapy-induced neurotoxicity.

## Abbreviations

BMI: Body Mass Index
BSA: Body Surface Area
DLN: Chemotherapy induced Dose-Limiting Neurotoxicity
DLT: Chemotherapy-induced Dose-Limiting Toxicities
ECOG PS: Eastern Cooperative Oncology Group Criteria Performance Status
FOLFOX: 5FU, leucovorin + oxaliplatin
HGS: Handgrip Strength
HR: Hazard Ratio
LBM: Lean Body Mass
mGPS: Modified Glasgow Prognostic Score
SD: Standard Derivation
UMA-CH: Ambulatory Digestive Cancer Unit
